# Development and internal validation of a therapeutic effect predictive model for myofascial pain syndrome

**DOI:** 10.3389/fneur.2026.1761946

**Published:** 2026-02-24

**Authors:** Xiumei Zhu, Wanquan Cheng

**Affiliations:** Department of Anesthesiology, Guannan County First People's Hospital, Lianyungang, China

**Keywords:** efficacy evaluation, individualized treatment, machine learning, myofascial pain syndrome, prediction model

## Abstract

**Background:**

Myofascial Pain Syndrome (MPS) is a common chronic pain disorder, and there are significant individual differences in its clinical efficacy. Currently, there is a lack of reliable prediction tools to guide individualized treatment decisions. This study aimed to construct and validate a prediction model based on clinical and biomarker data to evaluate the responses of MPS patients to different treatment regimens and optimize treatment strategies.

**Methods:**

A total of 340 MPS patients was retrospectively enrolled and randomly split into a training set (*n* = 238, 70%) and an internal validation set (*n* = 102, 30%). Baseline data (including pain characteristics, trigger point distribution, psychological status, and inflammatory markers) were collected. The patients received standardized treatment (including dry needling, physical therapy, and drug intervention), and the efficacy was evaluated after 8 weeks (primary outcome: pain relief ≥50%). Predictive factors were screened through multivariate logistic regression, and machine learning models (random forest, support vector machine, and K-nearest neighbor algorithm) were further constructed developed. Internal validation was performed using the Bootstrap resampling method. The model performance was evaluated by the area under the receiver operating characteristic curve (AUC), calibration curve, and decision curve analysis (DCA).

**Results:**

The final model included 6 key predictive factors (including disease duration, baseline pain intensity, Patient Health Questionnaire-9 depression score, pain catastrophizing score, interleukin-6, and high-sensitivity C-reactive protein levels). The AUC value of the support vector machine model reached 895 (95%CI: 0.840–0.950) in the training set and remained at a relatively high level of 0.873(95%CI: 0.794–0.953) in the validation set, and the calibration was good (Hosmer–Lemeshow test, *p* > 0.05). DCA showed that the model had a high clinical net benefit within the threshold probability range of 0.10–0.70.

**Conclusion:**

A MPS efficacy prediction model, which had good internal predictive efficacy and interpretability, integrating clinical, psychological and inflammatory indicators was successfully constructed and internally validated. In the future, multi-center external validation and model optimization are needed to further improve its clinical applicability and promotion value.

## Introduction

Myofascial pain syndrome (MPS) is a chronic musculoskeletal pain disorder characterized by myofascial trigger points. It has a high prevalence, seriously affecting patients’ quality of life and physical function, and is often accompanied by emotional disorders such as anxiety and depression (1). Currently, dry needling, physical therapy, and drug intervention are the core standardized treatment options for MPS recommended in current clinical guidelines, and most clinical institutions often adopt combined or sequential application of these modalities based on patients’ comprehensive conditions, aiming to inactivate trigger points and relieve pain (2). However, there are significant individual differences in patients’ responses to the same treatment, and this uncertainty in treatment efficacy severely restricts the precision of clinical treatment (3). At present, the evaluation of treatment efficacy mostly adopts retrospective analysis after treatment, lacking effective tools to predict individual treatment efficacy before treatment, resulting in the inability to achieve true individualized treatment (4). Studies have shown that the pathogenesis of MPS involves complex interactions among peripheral sensitization, central regulation, and neuro-immune-psychological factors (5). The differences in treatment response may be jointly affected by pain characteristics, psychological state, and inflammation levels. For example, pain catastrophizing cognition can amplify the pain experience, the depressive state may weaken the pain regulation ability, and the levels of inflammatory markers such as Interleukin-6 (IL-6) and high-sensitivity C-Reactive Protein (hs-CRP) may reflect different pathophysiological states (6, 7). Although a single indicator has certain predictive value for prognosis, research on constructing a comprehensive prediction model by integrating multi-dimensional variables is still relatively scarce. Machine learning techniques have unique advantages in processing complex medical data and establishing prediction models. This study intends to develop and validate a prediction model for the treatment efficacy of MPS based on patients’ pain characteristics, trigger point parameters, psychological assessment indicators, and inflammation marker levels at the baseline stage, using multivariate logistic regression combined with various machine learning algorithms, providing a basis for clinical individualized treatment and optimal allocation of medical resources.

## Methods

### Study subjects

A total of 340 patients with myofascial pain syndrome who visited the pain department and rehabilitation medicine department of our hospital from January 2020 to June 2025 and completed 8-week standardized treatment were retrospectively included. In this study, sample size calculation was first carried out. Based on the expected incidence of the primary outcome indicator (poor treatment efficacy), which was set at 35–40% [according to the previous data of our center and literature reports (8)], a scheme matching the core statistical methods (multivariate logistic regression and machine learning models) was adopted. The calculation was completed using the PASS 2021 software and verified using the “pwr” package in R 4.2.3. The significance level *α* was set at 0.05 (two-tailed), the test power 1–*β* was set at 80%, and a 10% dropout rate was considered. Finally, the minimum required sample size was 280 cases. The actual included sample size of 340 cases exceeded the minimum requirement, and its statistical power (1–*β* > 85%) was verified, meeting the statistical requirements for subsequent multivariate analysis and machine learning modeling.

Inclusion criteria: (1) Age ≥ 18 years; (2) Meeting the diagnostic criteria of MPS in the “Expert Consensus on the Diagnosis and Treatment of Myofascial Pain Syndrome in China,” with clear active myofascial trigger points and pain duration ≥3 months; (3) All receiving an 8-week standardized treatment protocol (including at least one of the core treatment methods such as dry needling, physical therapy, or drug intervention); (4) Completing a full set of baseline assessments before treatment, including pain characteristic assessment, trigger point examination, psychological state assessment, and inflammation marker detection; (5) Having complete visual analog scale scores and secondary outcome indicators after treatment for efficacy determination. Exclusion criteria: (1) Secondary pain caused by radiculopathy, rheumatological and immunological diseases, or acute trauma; (2) Comorbid fibromyalgia syndrome or other widespread pain diseases; (3) Comorbid severe mental illness or cognitive impairment, unable to cooperate to complete the assessment questionnaire; (4) Pregnant or lactating women; (5) Incomplete baseline assessment or 8-week follow-up data, unable to conduct efficacy grouping.

### Treatment types

The detailed parameters of each treatment type were as follows: (1) Physical therapy: Ultrasound therapy was adopted, with parameters of 1 MHz frequency, 1.5 W/cm^2^ intensity, 10 min per session, 3 times a week, for a total of 8 weeks; (2) Dry needling: Performed by physicians with more than 5 years of clinical experience, once a week, retaining the needle for 20 min each time, selecting 2–4 active trigger points per session based on physical examination, for a total of 8 weeks; (3) Drug therapy: Non-steroidal anti-inflammatory drugs (ibuprofen sustained-release capsules, 0.3 g each time, twice a day) combined with muscle relaxants (tizanidine hydrochloride tablets, 2 mg each time, three times a day), taken orally for 8 weeks. All patients strictly followed the treatment plan under the guidance of researchers.

The treatment allocation was based on the “Expert Consensus on the Diagnosis and Treatment of Myofascial Pain Syndrome in China” and the patients’ specific conditions: (1) patients with mild pain (VAS ≤ 5) and few trigger points (≤3) were first assigned to physical therapy; (2) patients with moderate pain (5 < VAS < 8) and obvious trigger points (3 < number ≤ 6) were assigned to dry needling combined with physical therapy; (3) patients with severe pain (VAS ≥ 8) or multiple trigger points (>6) were assigned to drug therapy (non-steroidal anti-inflammatory drugs + muscle relaxants) combined with dry needling and physical therapy. Patients’ willingness was fully considered during the allocation process. A statistical analysis of the treatment allocation distribution between the excellent and poor efficacy groups showed no significant difference (*p* > 0.05), indicating no confounding effect of treatment allocation imbalance.

Integrating these therapeutic modalities into a single predictive model can cover the mainstream clinical practice scenarios, making the model more universally applicable to different clinical settings, and this study strictly controlled the compliance of patients with treatment (physical therapy attendance rate ≥80%, drug/dry needling treatment compliance ≥85%) to reduce the impact of treatment implementation differences on the model.

### Data collection

In this study, multi-dimensional information was systematically collected through the hospital electronic medical record system, the clinical database of the pain department, and the special research follow-up system to construct a prediction model.

Patients’ basic information and clinical characteristics were collected, including: (1) demographic data: age, gender, height, weight, body mass index (BMI), occupation, and years of education; (2) living habits and medical history: smoking history (packs/year), alcohol consumption history (grams/day), exercise frequency (times/week), as well as previous medical history (such as cervical spondylosis, fibromyalgia), MPS duration (months), and drug use history; (3) pain characteristics: main pain location (such as neck, shoulder), pain side (left/right/bilateral), pain nature description (such as soreness, distending pain), and known inducing factors (such as acute injury, chronic strain).

Baseline assessment parameters before treatment were collected, covering: (1) pain characteristic parameters: baseline pain intensity at rest and during activity [visual analog scale (VAS) score, 0–10], average pain duration (hours/day), pain distribution range (count on the body map); (2) trigger point characteristics: number of active trigger points, main affected muscle groups, average tenderness threshold (kg/cm^2^); (3) psychometric indicators: including depression level [Patient Health Questionnaire-9 (PHQ-9)], anxiety level [Generalized Anxiety Disorder-7 (GAD-7)], and pain catastrophizing degree [Pain Catastrophizing Scale (PCS)]; (4) Inflammatory markers: serum levels of IL-6, tumor necrosis factor-alpha (TNF-α), and hs-CRP.

Finally, details of the treatment protocol (such as specific parameters of dry needling, physical therapy, or drug intervention) and treatment efficacy outcome data at 8 weeks after treatment were recorded. The primary outcome was the pain relief rate calculated based on the VAS score, and patients were grouped according to this, secondary outcomes included the improvement of the functional impairment index, changes in the tenderness threshold, etc. All data were uniformly managed through the electronic data collection system and were collected in a standardized manner by trained researchers to ensure the integrity and accuracy of the data. To ensure the reliability of VAS scores, each patient was assessed by the same trained pain department physician at both baseline and 8-week follow-up. The intraclass correlation coefficient (ICC) was used to evaluate the rater consistency, and the results showed ICC = 0.92 (95%CI: 0.88–0.95), indicating good consistency and reliability of the assessment results.

### Outcome definition

Referring to the assessment criteria for chronic pain treatment response in the International Association for the Study of Pain and the “Guidelines for the Diagnosis and Treatment of Myofascial Pain Syndrome in China,” based on the minimum clinically important difference (MCID) of the VAS and combined with the characteristics of the 8-week standardized treatment follow-up in this study, patients were divided into two groups.

Excellent treatment efficacy group: After 8 weeks of treatment, all of the following conditions were met: (1) primary efficacy criterion: The VAS pain score decreased by ≥50% compared with the baseline (reaching the MCID threshold); (2) stability requirement: The standardized treatment protocol was not discontinued during treatment due to intolerable adverse reactions or personal reasons; (3) compliance requirement: The attendance rate of physical therapy was not less than 80%, or the compliance record of drug/dry needling treatment was not less than 85% of the recommended protocol.

Poor treatment efficacy group: After 8 weeks of treatment, any of the following conditions were met: (1) efficacy not achieved: the VAS pain score decreased by <50% compared with the baseline; (2) symptom exacerbation: the VAS pain score showed no improvement or increased compared with the baseline; (3) treatment interruption: the patient withdrew from treatment early due to pain-related discomfort, adverse reactions, or lack of confidence in the protocol, and the VAS score at the time of withdrawal did not reach the excellent treatment efficacy standard.

All VAS assessments and secondary outcome indicators (such as the Neck Disability Index, tenderness threshold, etc.) were performed by pain department physicians or researchers who had received unified training through a standardized assessment process at two time points: before the start of treatment and 8 weeks after treatment. Efficacy determination (i.e., calculation of the VAS change value and final grouping) was independently completed in a double-blind manner by two researchers who were unaware of the patients’ other baseline clinical data and laboratory indicators. When there was a disagreement in grouping opinions, a third senior researcher in the research group made an arbitration confirmation according to the preset criteria. All clinical data and assessment results were entered and managed using the electronic data collection system to ensure the accuracy, integrity, and traceability of the data.

### Statistical analysis

Data analysis was completed using software such as SPSS 26.0, R 4.2.3, and Python 3.8.5. The Shapiro–Wilk test was used to verify the normality of measurement data. Measurement data conforming to the normal distribution (*p* > 0.05) were expressed as mean ± standard deviation (*x̄* ± *s*), and independent-samples *t*-test was used for comparison between groups; non-normally distributed data (*p* < 0.05) were expressed as median (quartiles), and the Mann–Whitney *U* test was used for comparison between groups. Missing values were filled using the median imputation method; continuous variables were standardized (*z*-score normalization) to eliminate the impact of dimension differences. Count data were expressed as number of cases (percentage) [*n* (%)], and the *χ*^2^ test or Fisher’s exact probability method was used for comparison between groups. In the training set, univariate analysis was first performed to screen out indicators with *p* < 0.05, then variables were compressed by Least Absolute Shrinkage and Selection Operator (LASSO) regression, and finally, multivariate logistic regression was used to determine independent influencing factors, and their odds ratios (OR) and 95% confidence intervals (CI) were calculated. Based on the results of multivariate analysis, machine learning models such as random forest, support vector machine, and K-nearest neighbor algorithm were further constructed using Python 3.8.5 and the sklearn library. The detailed implementation details were as follows: Random forest: n_estimators = 100, max_depth = 10, random_state = 42; Support vector machine: kernel = ‘rbf’, C = 1.0, gamma = ‘scale’, random_state = 42; K-nearest neighbor algorithm: n_neighbors = 5, weights = ‘distance’. The receiver operating characteristic (ROC) curve was drawn, and the area under the curve (AUC) was used to evaluate the prediction performance of the model. At the same time, the “rms” package in R software was used to construct a nomogram prediction model, and internal validation was carried out by the Bootstrap method (1,000 repeated samplings). The concordance index (C-index) was calculated to evaluate the discrimination ability, and the calibration curve was drawn to evaluate the calibration degree. Decision curve analysis (DCA) was used to evaluate the clinical application value of the nomogram by calculating the net benefit at different threshold probabilities. In addition, the “SHAP” library in Python was used to calculate SHAP values to evaluate the interpretability of the model from both global (feature importance ranking, contribution direction) and local (risk contribution decomposition of single-case patients) dimensions. Combined with the visualization function of the nomogram, the clinical interpretability of the model prediction results was achieved. A *p-*value < 0.05 was considered statistically significant.

## Results

### Comparison of general information of patients in the training set and validation set

A total of 340 patients with myofascial pain syndrome who completed 8-week standardized treatment were included in this study and randomly divided into a training set (*n* = 238) and a validation set (*n* = 102) at a ratio of 7:3. In the training set, there were 143 patients (60.1%) with excellent treatment efficacy and 95 patients (39.9%) with poor treatment efficacy. The validation set included 62 patients (60.8%) with excellent treatment efficacy and 40 patients (39.2%) with poor treatment efficacy. There were no statistically significant differences in baseline data such as age, gender, disease duration, pain characteristics (such as pain duration, distribution range), trigger point characteristics (such as number, tenderness threshold), psychometric indicators (such as PHQ-9, GAD-7, PCS scores), and inflammatory markers (such as IL-6, TNF-α, hs-CRP) between patients in the training set and the validation set (*p* > 0.05), indicating that the data set was evenly divided and had good comparability (Table 1).

**Table 1:** Comparison of general information of patients in the training set and validation set.

Indicators		Training set (*n* = 238)	Validation set (*n* = 102)	*t/χ^2^*	*P*
Age (years)		48.52 ± 13.24	47.83 ± 12.91	0.444	0.658
Gender	Male	89 (37.4%)	42 (41.2%)	0.431	0.511
	Female	149 (62.6%)	60 (58.8%)		
Disease duration (months)		22.37 ± 15.68	20.91 ± 14.32	0.807	0.420
Body mass index (kg/m^2^)		26.84 ± 4.10	27.15 ± 4.31	0.629	0.530
Baseline pain intensity (VAS, 0–10)		6.53 ± 1.82	6.34 ± 1.73	0.895	0.371
Pain duration (hours/time)		7.23 ± 4.52	6.94 ± 4.23	0.553	0.581
Pain distribution range (number)		2.81 ± 1.29	2.73 ± 1.22	0.533	0.595
Neck disability index (%)		41.24 ± 11.53	39.82 ± 10.74	1.062	0.289
Number of active trigger points (number)		4.23 ± 1.87	4.05 ± 1.76	0.828	0.409
Number of main affected muscle groups (number)		2.21 ± 0.83	2.14 ± 0.78	0.725	0.469
Average tenderness threshold (kg/cm^2^)		2.89 ± 0.73	2.92 ± 0.64	0.360	0.719
PHQ-9 depression score (points)		11.82 ± 4.53	11.24 ± 4.12	1.111	0.267
GAD-7 anxiety score (points)		9.53 ± 3.94	9.12 ± 3.71	0.895	0.372
Pain catastrophizing score (points)		28.43 ± 9.24	27.15 ± 8.83	1.186	0.236
IL-6 (pg/mL)		5.24 ± 2.38	5.03 ± 2.21	0.761	0.447
TNF-α (pg/mL)		3.82 ± 1.63	3.64 ± 1.50	0.955	0.340
hs-CRP (mg/L)		4.13 ± 2.23	3.92 ± 2.04	0.816	0.415
BDNF (ng/mL)		17.25 ± 5.83	17.91 ± 5.52	0.972	0.332

### Univariate analysis of influencing factors for the treatment efficacy of patients with myofascial pain syndrome

Among the 238 patients in the training set, 143 had excellent treatment efficacy. Among the 102 patients in the validation set, 62 had excellent treatment efficacy. Univariate analysis showed that in the training set, there were statistically significant differences between the patients with excellent treatment efficacy and those with poor treatment efficacy in terms of disease duration, baseline pain intensity, average tenderness threshold, PHQ-9 depression score, pain catastrophizing score, IL-6 level, and hs-CRP level (all *p* < 0.05) (Table 2).

**Table 2:** Univariate analysis of influencing factors for the treatment efficacy of patients with myofascial pain syndrome.

Indicators		Group with excellent treatment efficacy (*n* = 143)	Group with poor treatment efficacy (*n* = 95)	*t/χ^2^*	*P*
Age (years)		47.82 ± 12.95	49.61 ± 13.68	1.021	0.308
Gender	Male	56 (39.2%)	33 (34.7%)	0.477	0.490
	Female	87 (60.8%)	62 (65.3%)		
Disease duration (months)		18.52 ± 10.41	27.85 ± 17.37	5.177	0.001
Body mass index (kg/m^2^)		26.51 ± 3.98	27.32 ± 4.26	1.495	0.136
Baseline pain intensity (VAS, 0–10)		5.98 ± 1.65	7.38 ± 1.72	6.303	0.001
Pain duration (hours/time)		6.95 ± 4.23	7.65 ± 4.93	1.170	0.243
Pain distribution range (number)		2.69 ± 1.24	2.94 ± 1.33	1.480	0.140
Neck disability index (%)		40.85 ± 10.72	43.38 ± 12.25	1.684	0.094
Number of active trigger points (number)		4.05 ± 1.76	4.50 ± 2.01	1.824	0.069
Number of main affected muscle groups (number)		2.15 ± 0.79	2.30 ± 0.88	1.370	0.172
Average tenderness threshold (kg/cm^2^)		3.12 ± 0.68	2.85 ± 0.70	1.063	0.157
PHQ-9 depression score (points)		10.15 ± 3.82	14.72 ± 4.51	8.403	0.001
GAD-7 anxiety score (points)		8.82 ± 3.55	9.78 ± 4.28	1.880	0.061
Pain catastrophizing score (points)		25.18 ± 8.15	34.15 ± 8.72	8.085	0.001
IL-6 (pg/mL)		4.52 ± 1.95	6.73 ± 2.05	8.389	0.001
TNF-α (pg/mL)		3.65 ± 1.45	4.01 ± 1.85	1.678	0.095
hs-CRP (mg/L)		3.58 ± 1.87	5.28 ± 2.11	6.523	0.001
BDNF (ng/mL)		17.58 ± 5.92	16.78 ± 5.68	1.038	0.301

### Multivariate logistic regression analysis for predicting treatment efficacy in MPS patients

**Figure 1:**
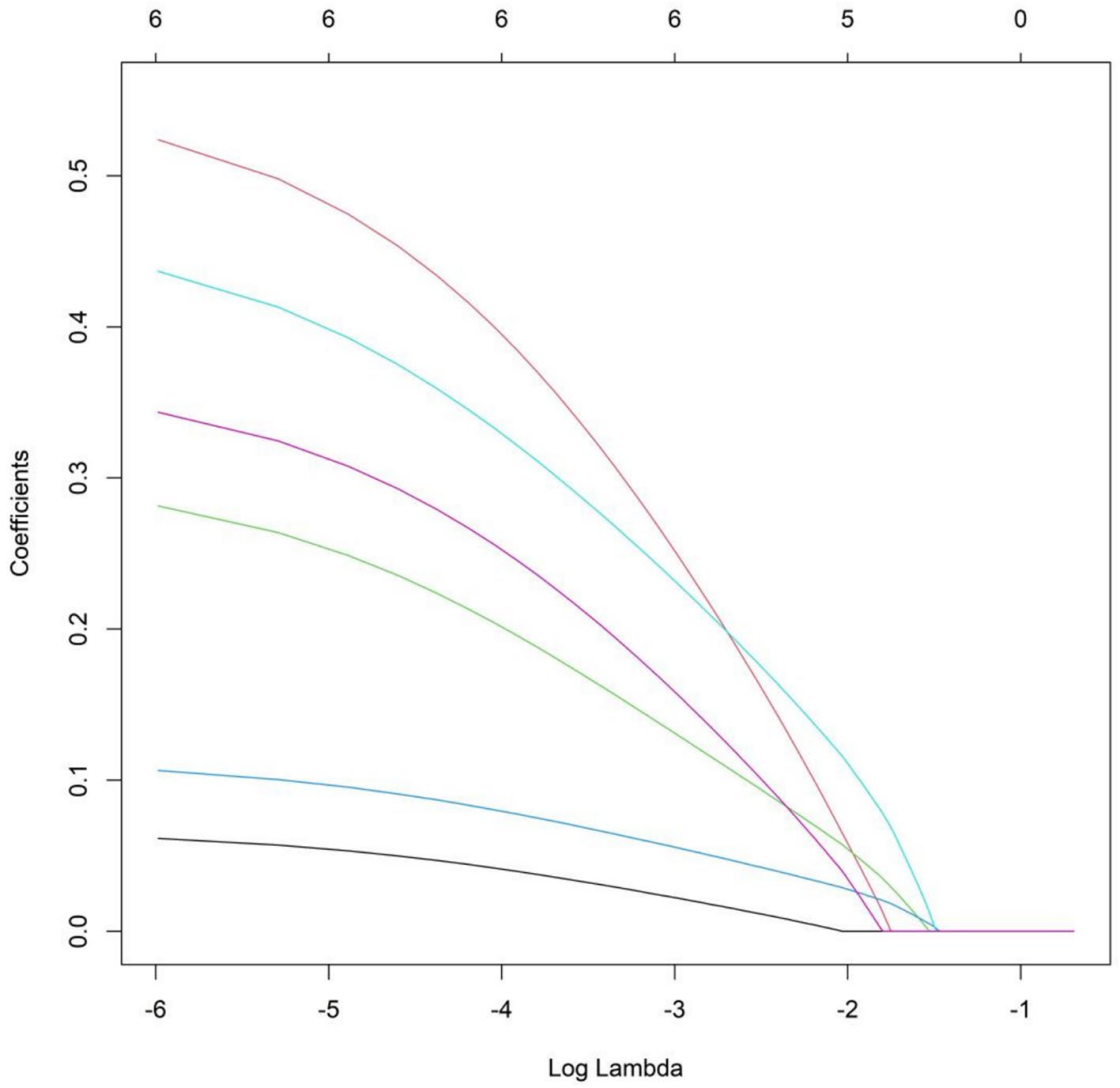
Variable selection using the LASSO regression model.

The therapeutic efficacy of myofascial pain syndrome in patients was used as the dependent variable (1 = poor efficacy group, 0 = excellent efficacy group). Six indicators with statistical significance in the univariate analysis (disease duration, baseline pain intensity, PHQ-9 depression score, pain catastrophizing score, IL-6, and hs-CRP levels) were all included in the LASSO regression for variable screening (Table 3). The optimal variables were selected using 10-fold cross-validation and the *λ*-1se criterion (Figure 1). Finally, all 6 predictive variables were retained and included in the multivariate logistic regression model. The results of the multivariate logistic regression analysis showed that disease duration, baseline pain intensity, PHQ-9 depression score, pain catastrophizing score, IL-6, and hs-CRP levels were independent risk factors for poor therapeutic efficacy (all *p* < 0.05) (Table 4).

**Table 3:** Variable assignment table.

Variable	Meaning	Assignment
X1	Disease duration	Continuous variable
X2	Baseline pain intensity	Continuous variable
X3	PHQ-9 depression score	Continuous variable
X4	Pain catastrophizing score	Continuous variable
X5	IL-6	Continuous variable
X6	hs-CRP	Continuous variable
Y	Therapeutic efficacy	1 = Poor efficacy group, 0 = Excellent efficacy group

**Table 4:** Multivariate logistic regression analysis of the therapeutic efficacy of myofascial pain syndrome in patients.

Indicators	*β*	SE	Wald	P	OR	95%CI
Disease duration	0.060	0.019	10.503	0.001	1.062	1.024 ~ 1.102
Baseline pain intensity	0.404	0.140	8.306	0.004	1.498	1.138 ~ 1.971
PHQ-9 depression score	0.279	0.064	19.347	0.001	1.322	1.168 ~ 1.971
Pain catastrophizing score	0.139	0.031	20.736	0.001	1.149	1.083 ~ 1.220
IL-6	0.505	0.118	18.190	0.001	1.658	1.314 ~ 2.091
hs-CRP	0.431	0.119	13.162	0.001	1.539	1.219 ~ 1.943

### Performance evaluation of machine learning models

**Figure 2:**
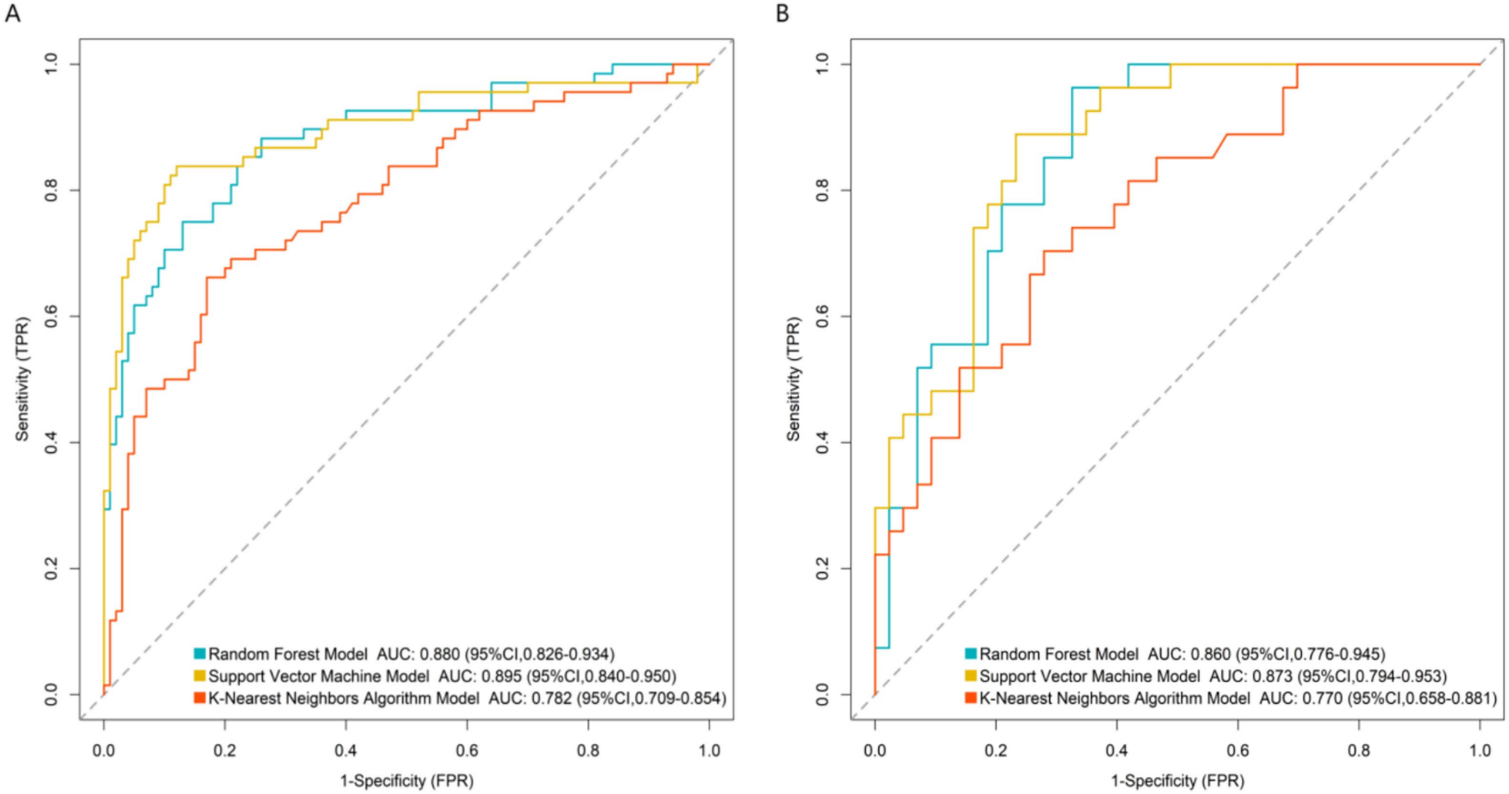
Receiver operating characteristic curves in the training set **(A)** and the validation set **(B)**.

**Figure 3:**
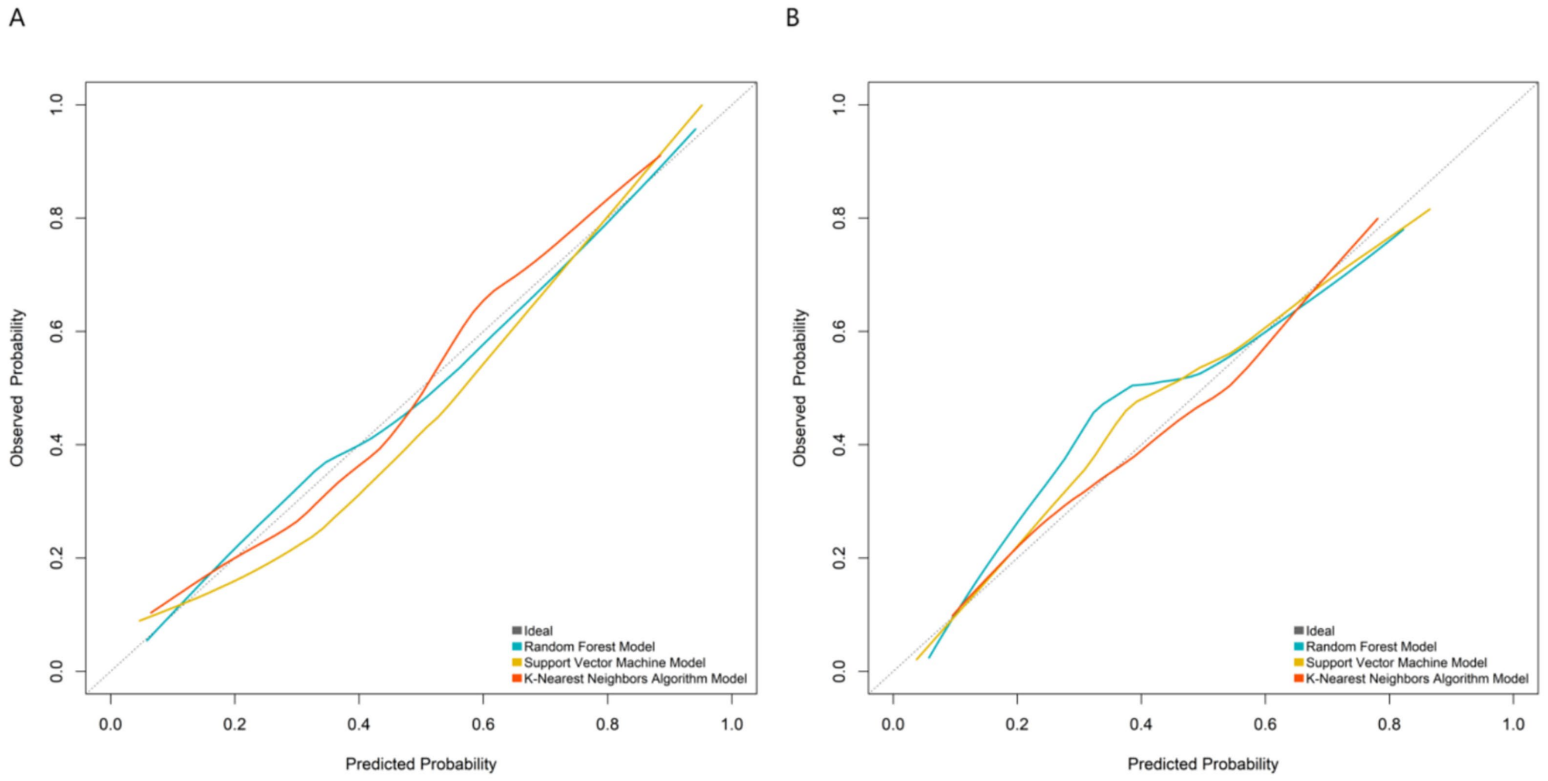
Calibration curves in the training set **(A)** and the validation set **(B)**.

**Figure 4:**
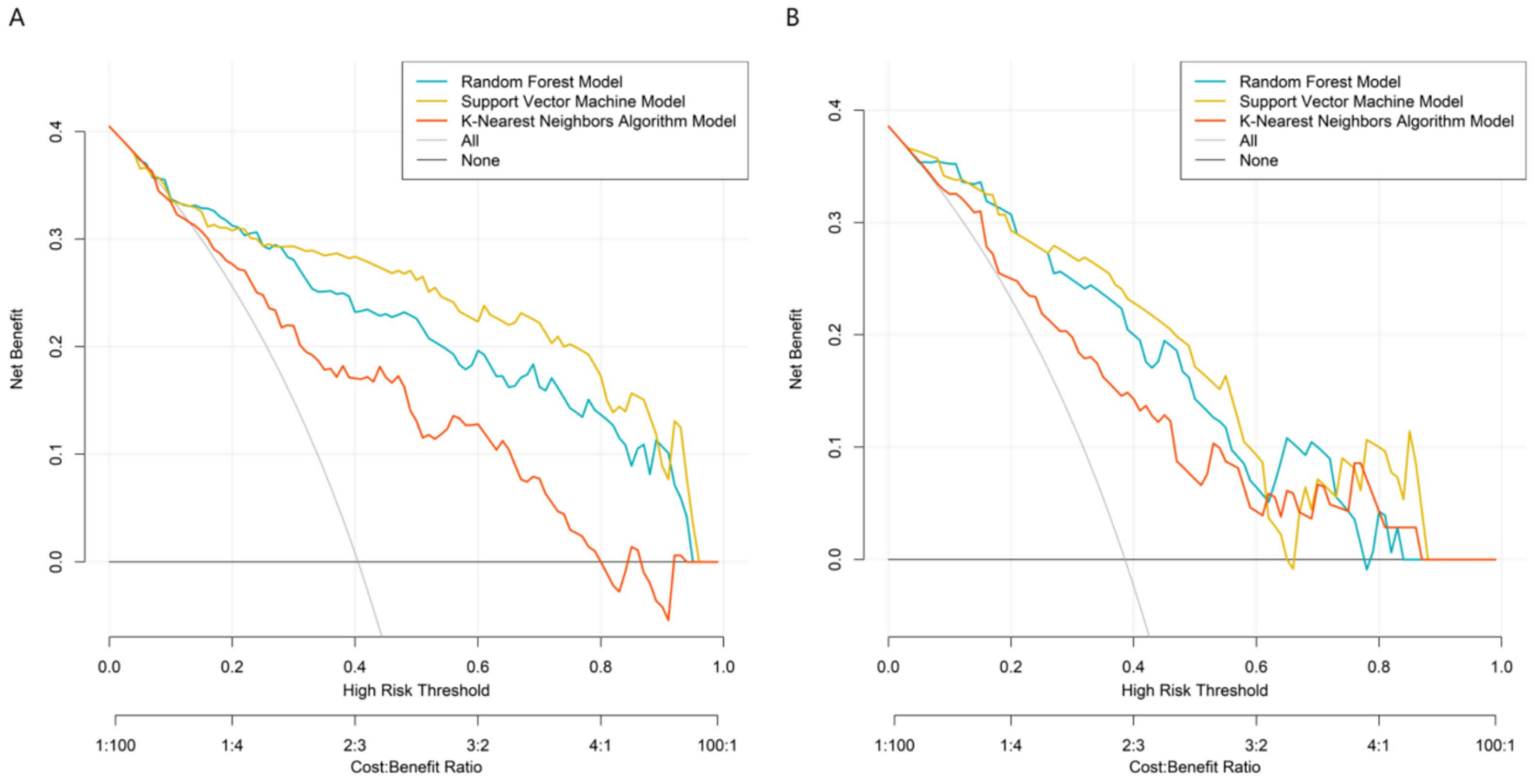
Decision curves in the training set **(A)** and the validation set **(B)**.

By systematically comparing the performance of each model on the training set and the validation set, the results in Figure 2 showed that the AUC value of the support vector machine model reached 0.895 (95%CI:0.840–0.950) in the training set and remained at a relatively high level of 0.873 (95%CI: 0.794–0.953) in the validation set, which were significantly better than those of other comparison models (Random forest: 0.880/0.860, K-nearest neighbor algorithm: 0.782/0.770). Therefore, the support vector machine model was determined as the best prediction model in this study. This result proves that the support vector machine model has a stable and excellent ability to distinguish patients with poor therapeutic efficacy of myofascial pain syndrome. Further analysis of the calibration curve in Figure 3 revealed a high degree of consistency between the predicted probability of the model and the actual observed risk, with the curve closely fitting the diagonal, indicating good prediction calibration and reliable prediction results. In addition, the DCA in Figure 4 confirmed that within a wide threshold probability range of 0.10–0.70, the clinical net benefit of applying this prediction model was always significantly higher than that of the extreme strategies of “treating all” or “not treating all”, demonstrating its outstanding clinical practical value. In summary, the support vector machine prediction model developed based on multi-dimensional clinical indicators in this study has good prediction accuracy, calibration, and clinical applicability.

### Interpretability evaluation of model prediction results

**Figure 5:**
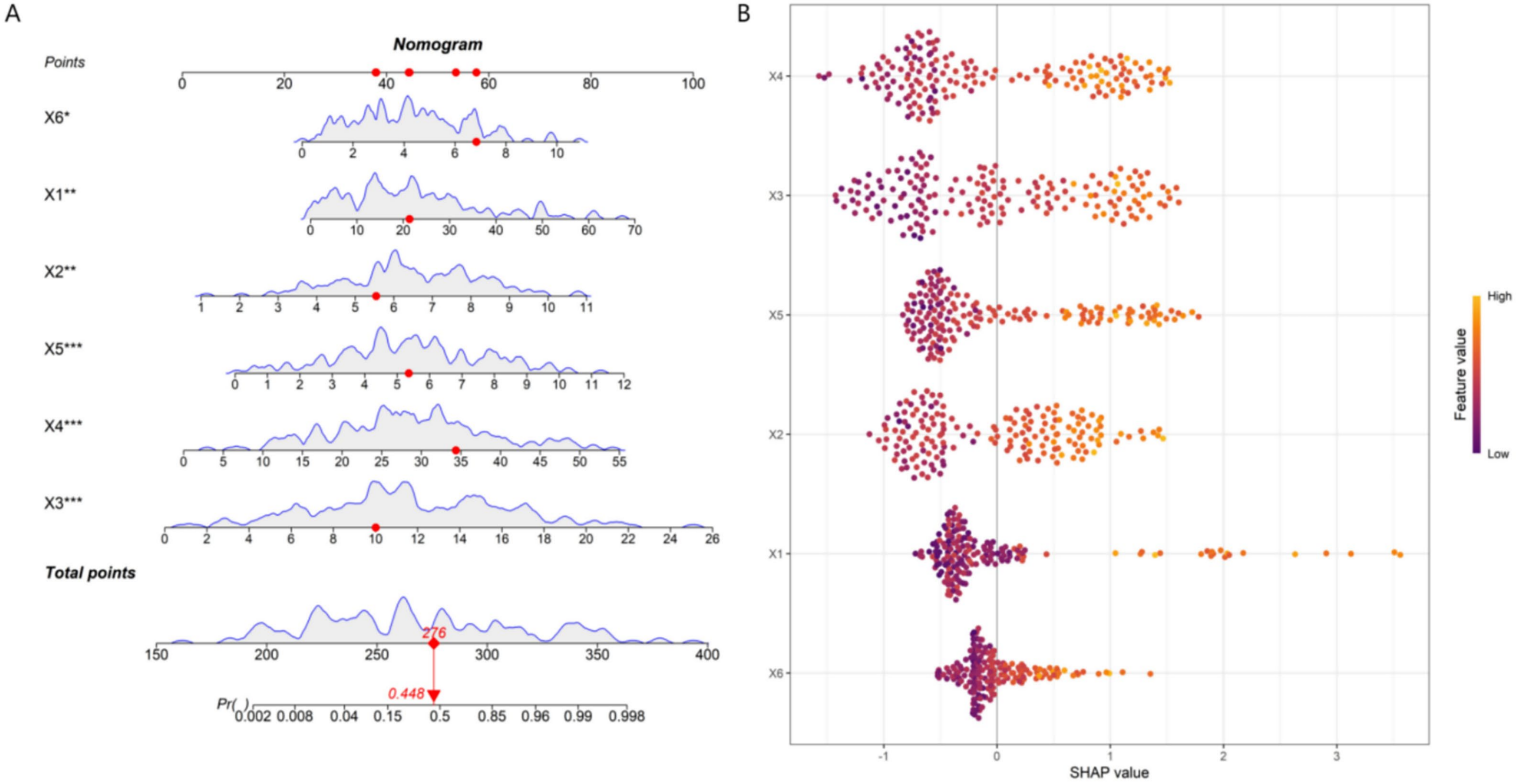
Model interpretability analysis (**A**: Fancy nomogram; **B**: SHAP feature importance plot). X1, Disease duration; X2, Baseline pain intensity; X3, PHQ-9 depression score; X4, Pain catastrophizing score; X5, IL-6 level; X6, hs-CRP level.

Based on the six core predictive variables screened by the multivariate Logistic regression analysis (disease duration, baseline pain intensity, PHQ-9 depression score, pain catastrophizing score, IL-6, and hs-CRP levels), this study used the support vector machine algorithm to construct a nomogram model for predicting the therapeutic efficacy of MPS. As shown in Figure 5, this nomogram visually demonstrated the contribution degree and direction of each clinical feature to the treatment outcome. The model results showed that disease duration (X1), baseline pain intensity (X2), PHQ-9 depression score (X3), pain catastrophizing score (X4), IL-6 (X5), and hs-CRP (X6) were all independent risk factors for poor therapeutic efficacy, and an increase in their values would significantly increase the risk of treatment ineffectiveness. SHAP analysis further quantified the relative importance of each feature, and the order of influence from large to small was: PHQ-9 depression score (X3), IL-6 level (X5), pain catastrophizing score (X4), baseline pain intensity (X2), disease duration (X1), and hs-CRP level (X6). Among them, the PHQ-9 depression score and IL-6 level made the most prominent positive predictive contributions to treatment ineffectiveness. Although disease duration and baseline pain intensity were also risk factors, their relative influence weights were slightly lower than those of psychological and inflammatory indicators. The results of the SHAP analysis were highly consistent with the conclusions of the multivariate logistic regression, confirming the core position of psychological factors and inflammatory markers in predicting the therapeutic efficacy of MPS.

## Discussion

Myofascial Pain Syndrome, as a common chronic musculoskeletal pain disorder, exhibits significant heterogeneity in treatment response. Currently, there is a lack of effective tools in clinical practice to accurately predict individual treatment efficacy before treatment. In this study, a support vector machine model for individualized prediction of MPS treatment efficacy was innovatively developed and validated by integrating clinical pain characteristics, psychological states, and inflammatory biomarkers in a multi-dimensional manner. The model demonstrated excellent and stable predictive performance in both the training set and the validation set. The robustness of the predictive variables and the optimal performance of the model were ensured through comparisons among LASSO regression, multivariate logistic regression, and various machine-learning algorithms. More importantly, in-depth interpretability analysis of the model was conducted by combining nomograms and SHAP analysis. This not only quantified the relative importance of each predictor but also clarified the risk relationship between them and poor treatment outcomes, providing new perspectives and evidence for understanding the mechanism of treatment efficacy differences in MPS and achieving precise clinical intervention.

The six core predictors finally identified in this study profoundly reflect the essence of MPS as a complex pathophysiological process involving peripheral sensitization, central regulation, and systemic inflammatory responses. Among them, the PHQ-9 depression score and IL-6 level were proven to be the most influential predictors. Depressive states are often accompanied by weakened function of the descending pain inhibitory pathway and pain catastrophizing cognition, which significantly amplify pain perception and weaken treatment response (9). Meanwhile, as a key pro-inflammatory cytokine, IL-6 can directly act on the peripheral and central nervous systems, promoting peripheral and central sensitization and serving as an important molecular basis for maintaining the chronic pain state (10). The high contributions of these two factors highlight the core role of the neuro-psychological-immune axis in the chronicity and treatment resistance of MPS.

Notably, the pain catastrophizing score was also identified as a key independent risk factor. Pain catastrophizing reflects the cognitive-emotional state of patients with negative magnification, rumination, and helplessness regarding pain. It not only directly exacerbates the pain experience but also leads to avoidance behavior and decreased treatment compliance, thus forming a vicious cycle that hinders recovery (11–13). This model confirmed that pain catastrophizing cognition itself is an important driving factor for poor treatment efficacy after excluding the influence of depressive mood.

In addition, baseline pain intensity and disease duration, as classic clinical variables, had their predictive values confirmed in this model. Higher baseline pain intensity and longer disease duration often imply more stubborn pain memory pathways and more significant structural changes, correspondingly increasing the treatment difficulty (14, 15). The elevated level of hs-CRP, a stable marker of systemic inflammatory status, further corroborates that low-grade systemic inflammation is an important background for the pathophysiology and treatment efficacy differences of MPS (16–18).

The core innovation of this study lies in breaking through the limitations of traditional prediction mainly relying on clinical manifestations and achieving multi-dimensional integration of clinical, psychological, and biomarker data. Our prediction model indicates that the treatment effect of MPS is not determined by a single factor but is the result of the combined action of local pain characteristics (intensity, duration), central cognitive-emotional processing (depression, catastrophizing), and the systemic pathological environment (inflammation). This perfectly confirms the bio-psycho-social model theory of MPS (19, 20). In real-world clinical practice, clinicians can input patients’ baseline data (disease duration, baseline pain intensity, PHQ-9 score, pain catastrophizing score, IL-6 and hs-CRP levels) into the model to obtain the probability of poor treatment efficacy. For patients with a high predicted probability (e.g., >0.5), clinicians can adjust the treatment strategy in advance, such as increasing the intensity of psychological intervention or adding anti-inflammatory interventions, which provides an objective quantitative basis for clinical individualized treatment selection beyond standard clinical assessment.

In terms of methodology, this study combined the advantages of traditional statistical methods and modern machine-learning techniques. First, feature selection was performed through LASSO regression, and then the independent effects and risk ratios of each factor were clarified through multivariate logistic regression. Finally, a model with stronger predictive performance and non-linear fitting ability was constructed using the support vector machine algorithm (21). This combined strategy not only ensured the statistical rigor of the model but also improved the prediction accuracy (22). Moreover, the application of SHAP interpretability analysis was a highlight of this study. It objectively quantified the contribution ranking of each feature and clearly showed the positive and negative contribution directions of each feature to individual prediction results. This “white-box” processing greatly enhanced clinicians’ trust and understanding of the model decision-making process, promoting the transformation of the prediction model from “predictive performance” to “clinically reliable.”

This study has several limitations. First, the study only conducted internal validation through Bootstrap resampling and was unable to perform external multicenter validation due to resource constraint. This limits the generalizability of the model to other populations and clinical institutions, as the baseline characteristics of patients, treatment protocols, and medical practice patterns may vary across different centers. In future research, we will actively seek multi-center collaboration opportunities to conduct prospective external validation, aiming to verify the model’s external applicability and further optimize it based on multi-center data. Second, although major inflammatory diseases were excluded, low-grade chronic inflammation related to obesity and metabolic syndrome may affect the levels of IL-6 and hs-CRP, which are potential unmeasured confounding factors. We will include these factors as covariates in future model optimization to reduce the impact of confounding. Third, all predictive variables were derived from baseline assessments, and dynamic change factors during the treatment process were not included. In the future, the combination of mobile health technology can be explored to construct a dynamic prediction model. Finally, this study integrates multiple standardized treatment modalities into a single model, and although univariate analysis shows no significant difference in treatment modality distribution between the excellent and poor efficacy groups, treatment heterogeneity may still have a potential impact on the model’s predictive performance for specific treatment responses. The lack of stratified analysis by treatment modality limits the interpretation of the model in guiding specific treatment selections.

A prospective multicenter external validation study is planned in the next phase. This study will adopt a prospective cohort design and recruit MPS patients from 3 to 5 tertiary hospitals in different regions of China (covering northern, southern, eastern and central China). According to AUC = 0.895, *α* = 0.05, power = 0.8 and 10% dropout rate, the planned sample size was 300. Data collection will include the same baseline measures and 8-week efficacy outcomes as in this study, and the external generalization of the model will be assessed by means of AUC, calibration curve, and decision curve analyses. In addition, future studies will establish special prediction models for specific treatment methods, including dry acupuncture therapy alone, physical therapy alone, drug therapy alone, and different combination therapies. For each specific treatment, we will collect detailed treatment parameters (such as dry needle frequency, physical therapy intensity, drug dose, etc.) to construct a specialized model, so as to help clinicians obtain more accurate prediction of therapeutic effect according to the proposed treatment plan and further improve the clinical application value and accuracy of the model.

In conclusion, this study successfully developed and internally validated a prediction model for the treatment efficacy of MPS, which integrates clinical, psychological, and inflammatory indicators and has good internal predictive performance and interpretability. The model can provide a preliminary objective basis for clinical individualized treatment selection as a clinical decision-making auxiliary tool. However, this study has certain limitations, including single-center design, lack of external validation, and potential unmeasured confounding factors, which limit the generalizability of the model. Therefore, the findings of this study should be interpreted with caution, and the model should be used in combination with clinical practice and patient-specific conditions. Future external multicenter validation studies and model optimization are needed to further improve the clinical utility and generalizability of the model.

## Data Availability

The original contributions presented in the study are included in the article/supplementary material, further inquiries can be directed to the corresponding author.
